# Continuous Local Infection

**Published:** 1904-02-15

**Authors:** Rickman J. Godlee


### CONTINUOUS LOCAL INFECTION.

BY RICKMAN J. GODLEE, M.S., LONDON, F.R.C.S., ENGLAND.

Published in "London Lancet."

One of the most important diseases of this class is *pyorrhœa alveolaris* and I make no scruple in referring to it although it is now rather prominently before the profession and the public and I have already myself published something upon it. It is important, first, because it is so common and so insiduous; secondly, because its effects are so pernicious and so varied; and thirdly, because it is so difficult to cure that our friends, the dentists, are apt to give it up as a bad job. Spongy gums seem to be such a trivial complaint and the inconvenience is to many people—people who are not very particular—so slight that they seldom, unless the importance of it is pointed out by the medical attendant or the dentist, think it worth while to apply for treatment. The people who never wash their teeth seem to suffer but little from it, even when it is far advanced and their mouths are in a foul condition, though what their sensations must be I find it hard to conjecture. In our own walk of life the complaints usually made are: (1) that the gums bleed when the teeth are washed or when they are sucked; (2) that there is a disagreeable taste in the mouth; and (3) sometimes, but by no means always, that there is a large amount of expectoration which may have the appearance of pus mixed with saliva or be more or less blood-stained. The patients will often tell you that it is not only a highly-offensive taste but an offensive smell, though of this they are often quite unconscious. Any one, however, who has once recognized the very disagreeable sickly odor which is associated with the disease has, unfortunately, only too often the opportunity of recognizing its presence among his own acquaintances. It is often asserted most positively that this expectoration comes from the back of the nose or the throat or is coughed up from the lung and patients are only convinced that they have been mistaken when the disease has been cured or placed in abeyance. Occasionally the saliva or bloody fluid runs out from the mouth at night on to the pillow, and this when it does occur is almost pathogenomic of the disease. If the expectoration be allowed to settle, the deposit has a very close superficial resemblance to pus. It does, indeed, contain a certain amount of pus, but by far the greater part of it consists of squamous epithelium, which should at once suggest its source. It is perhaps not generally known that a small amount of fluid of this sort—I mean one looking like pus mixed with saliva—may be obtained by any one, however clean his mouth, by sucking for a few minutes at his gums with the tongue. The signs of the disease are not very obvious if the patient is particular and brushes his teeth frequently; perhaps only a bright red line at the edge of the slightly-retracted gums. I particularly want to emphasize the fact that such appearances are consistent with a very advanced stage of the disease. A very fine probe will very likely, even when there is nothing more to be seen, pass a long way between the separated gums and the fangs of the teeth, and it is from these pockets that the noxious material is being constantly expelled into the mouth. In advanced cases when the patient takes no care of his mouth there is no difficulty in making a diagnosis. The extensive retraction of the gums which are obviously spongy and probably bleeding, the yellow line of fur and pus, the absorption of the alveolus and exposure of the fangs covered with tartar, the gradual loosening of the teeth and the stinking mouth are quite unmistakeable.

Without attempting to summarize all that is known of pyorrhœa, and before coming to the reason for including it in this paper, there are two are three things more to be said. 1. It very often occurs in members of the same family, whether because it is hereditary, or infectious, or because the habits of the individuals are the same, I do not know. 2. The fangs of the teeth, where they are surrounded by the pockets, are covered with tartar, and it is impossible to cure the disease without eradicating these deposits. It is here that the chief difficulty of treatment comes in. The tartar is of two kinds, true tartar or salivary calculus consisting of phosphate and carbonate of lime with ptyalin and animal matter, and black tartar, dark in color and of dense consistence. The relation of the tartar to the disease has been much discussed. By some it is supposed to cause inflammation of the gums by its presence, and so to start the ulcerative process. By others it is supposed to be a secondary affair. For us it is important to remember that black tartar is found on the fangs of the teeth right down to the bottom of the pockets. It was on the removal of this tartar that Dr. Riggs, of Hartford, Connecticut, very strongly insisted in what was really the first rational suggestion for the treatment of the disease, and I do not altogether grudge his memory the name of "Riggs' disease," by which for long it was known. His treatment was by special powerful scarifiers and is said to have been painful but for a time successful. 3. The disease usually starts in young adult life and if unrelieved will almost inevitably lead to premature loss of the teeth. 4. Controversy is still maintained as to whether or not it is often or ever caused by the gouty diathesis, but the balance of evidence appears to be against this suggestion.

Now I will ask you to consider what it must be to be always swallowing day and night, year in and year out, these septic organisms and their products. I can imagine an objector saying that the stomach is well able to give a good account of micro-organisms and that a mouthful of cheese contains more living inhabitants than does the continent of Europe, or urging that if the disease is so common we ought to see more of its results, or again that the human body is after all a joint-stock concern and gets along very well with its associated organisms and, in fact, might be rather in difficulties without them. In order to answer these criticisms let us try to realize the actual state of things as far as modern investigations enable us to do so. An admirable work on "The Mycology of the Mouth," by Kenneth Weldon Goadby, has appeared this year, in which he gives with careful detail his own observations as well as those of previous observers, of whom the best known are Miller and Gallippe. It is there stated that the micro-organisms seen on direct examination may be tabulated as follows: (1) cocci, generally in diplococci, and massed around the epithelial cells in clumps; (2) thick bacilli, generally jointed and often showing considerable irregularity in their staining characters; (3) thick bacilli with pointed ends and somewhat of the shape of a bean-pod (they frequently show a division in the center and appear as two elongated triangles with the bases opposed); (4) various fine bacilli, 0.5*m* and under in width, often exhibiting an irregular, beaded marking, especially well marked in the larger threads which may be of great length; (5) spiralla spirochætœ, and comma-shaped bacilli, all showing marked motility in the hanging drop; (6) various yeast forms, sometimes with a partially-developed mycelium; (7) streptothrix threads, generally showing well-marked clubs; (8) masses of bacilli associated with the threads, some jointed in chains, others free and often massed in clumps; some of them exhibit polar staining. Many of these organisms obtained in pure culture are pathogenic to animals, but it is remarkable that the best observers agree that the common pus cocci only occur in about 10 percent of the cases examined. Cocci, indeed, are found in all cases, but it is supposed that they are not precisely the same as the pus cocci. Be that as it may, it is clear that a vastly larger number of pathogenic organisms, and probably in a much more active state than normal, are constantly being swallowed. The stomach no doubt can deal with a good many organisms by virtue of the hydrochloric acid in the gastric juice. But there are many organisms which it will not kill—

such, for example, as the typhoid bacillus—and it has not been ascertained how far the pathogenic organisms of the mouth are affected by it. They come down in great numbers and the resisting power of numbers is greater than the resisting powers of a few; in other words, a concentrated dose is more effectual than a dilute one. They come down too, at all times of the day, both when the stomach is full and contains hydrochloric acid and when it is empty and contains little or none; and Dr. William Hunter considers that he has demonstrated that in cases where there is profuse purulent discharge into the mouth the pus cocci become permanent residents in the stomach after producing what he calls septic gastritis. Lastly, it is impossible to say why one person suffers from septic poisoning and another escapes, just as it is in the case of the exhibition of other toxins, whether septic or not. It is the personal equation which we do not understand. It is the amount of resistance that is greater in one individual than in another or is greater in the same individual at one time than at another, depending on the state of his bodily health at the time.

I now pass on to enumerate the diseases which have been supposed to be caused by pyorrhœa and other forms of oral sepsis, a long account of which is to be found in Hunter's paper and in the writings of others. They include septic fever, rashes, and profound septicæmia, purpuric hæmorrhages and bleedings from the gums, toxic neuritis, septic gastritis, stomatitis, pharyngitis, and tonsillitis, ulcerative endocarditis, pernicious anæmia, chronic dyspepsia, bronchiectasis, etc. This is a sufficiently formidable list, but not, I am sure, a complete one.

I desire, however, to draw attention not only to the diseases which pyorrhæa may cause, but also to those which it may simulate. I reported three instructive cases in the paper above mentioned, and to these, with one or two others, I will briefly refer.

Case 1.—The patient was a woman, aged 45 years, with a long history of previous illness dating back to 1881, including a miscarriage, a bad confinement, uterine displacements, vaginal discharge, floating kidney, shivering fits and headaches, and five or six attacks of influenza with chest and bronchial troubles. The expectoration began in 1897, and was at first green and then bloody and offensive. The diagnoses made were: pleurisy followed by a small empyema, pleurisy followed by bronchiectasis, and sub-diaphragmatic abscess connected with the right kidney and bursting into the lung. But there were no abnormal physical signs except slight retraction of the right posterior base with lessened movement, some increased resistance on percussion, and weak breath sounds. Her principal complaint was the extremely offensive expectoration, amounting to five or six ounces in the twenty-four hours, not exactly coughed up but hacked up from the back of the throat, a foul taste, and breath offensive to others. The teeth were very carefully attended to and only showed the red line and slight retraction of gums to which reference has been made, but the pockets were numerous and deep. All her troubles were removed by the effectual treatment of the gums. Her daughter suffers from the same complaint.

Case 2.—The patient was a woman, aged 25 years, who had a well-marked attack of right pleurisy in March, 1899. Expectoration began at the time of this illness, and became bloody six months later, and was always offensive. It was brought up in the same way as in the last case. She had lost flesh and perspired at night, and was very anxious about her state, and especially about the foul taste in her mouth and offensive smell of her breath. A few physical signs resulting from the old pleurisy remained at the right base, and I was requested to open a supposed abscess of the lung in this situation. She had very marked pyorrhæa, and was at once relieved on treating the gums. Her sister suffered from pyorrhœa.

Case 3.—The patient was a man, aged 66 years, who had very marked pyorrhœa round his few remaining teeth.

He had suffered from three or four mild attacks of diarrhœa in quick succession when he was seized with an acute attack of glossitis and stomatitis with profuse and offensive salivation. The gums became much more swollen and the suppuration copious. The tongue was enlarged and thickly coated. Superficial ulceration appeared at several spots where the teeth impinged on the lips and under surfaces of the tongue. There was for a few days considerable fever. He also rapidly improved under vigorous treatment of the gums.

Case 4.—Dr. Martin supplied me with notes of a case which was supposed to be cancer of the stomach depending on the same cause.

Case 5.—A woman was sent with obscure abdominal troubles which it was thought might be relieved by fixing the movable right kidney, though her symptoms depended only on pyorrhœa.

There are, however, other reasons why pyorrhœa is important to surgeons. I need not remind my surgical colleagues of the importance of treating it thoroughly before undertaking any operation upon the mouth nor how much the mortality of operations on the stomach, the tongue and the jaws has been reduced since a careful toilet of the mouth has been recognized as an essential preliminary. I have always a supply of tooth-brushes and Miller's excellent mouth wash at hand in my wards for all comers and direct the sister to inquire into the state of the patients' mouths and to deal out the tooth-brushes when necessary. Then I have seen quite a few where the disease has been mistaken for chronic pharyngitis, diseases of the antrum, and other inflammatory diseases of the mouth and naso-pharynx. Lastly, we must examine ourselves periodically and see whether the subjects of this disease, for it must not be forgotten that we become dangerous to the community if we have not clean mouths. A short-sighted or over-eager surgeon may spoil the effects of the elaborate antiseptic preparation of his patients by breathing or coughing on to the surface of a wound; an irritable or didactic one may by injudicious speaking during an operation positively put his patient's life in danger; in fact, I have more respect for the practice of some surgeons who always antisepticize their mouths before an operation and wear a mask and rigidly refrain from speaking during its performance than for some of those elaborate details which almost seem like bowing down to, and worshipping, a fetich.

One word must be said about the treatment. It does not, of course, rest with us but with the dentists who have carefully and repeatedly to clear away the tartar from the fangs and then to attack the organisms contained in the pockets either by syringing or packing, using such antiseptics as peroxide of hydrogen, chloride of zinc, or essentials oils, and sometimes freely slitting up the pockets in order to gain access to the mischief and give free exit to the pus. I regret to say that some dentists I have sent patients to have treated the notion that pyorrhœa could possibly cause such symptoms as I have described as moonshine or have plainly told the patients that they are incurable, or have advised the extraction of all the teeth. The last is certainly effectual, but is is rather a strong measure, and I know from ample experience that with patience and perseverance on the part of the patient very much may be done without proceeding to the *ultima ratio*. But it must be added that the problem of the effectual treatment of Riggs' disease amongst the poor has not yet been solved, and I would earnestly commend it to the attention of scientific dentists. I suggest that it will be found in making freer incisions into the gums, so that the pockets can be done away with, treating them, in fact, like ordinary abscesses, and then supplying the patients with good, hard tooth-brushes and some cheap but efficient oral-bactericidal mouth wash. It is not too much to hope that in otherwise explained cases of chronic septic poisoning we shall all get into the habit—that is the important thing—of critically examining the gums and insisting upon the treatment of this most insiduous complaint.—*Dental Record.*

